# Quantifying the effect of human population mobility on malaria risk in the Peruvian Amazon

**DOI:** 10.1098/rsos.211611

**Published:** 2022-07-20

**Authors:** Gabriel Carrasco-Escobar, Jose Matta-Chuquisapon, Edgar Manrique, Jorge Ruiz-Cabrejos, Jose Luis Barboza, Daniel Wong, German Henostroza, Alejandro Llanos-Cuentas, Tarik Benmarhnia

**Affiliations:** ^1^ Health Innovation Lab, Institute of Tropical Medicine ‘Alexander von Humboldt’, Universidad Peruana Cayetano Heredia, Lima, Peru; ^2^ Instituto de Medicinal Tropical Alexander von Humboldt, Universidad Peruana Cayetano Heredia, Lima, Peru; ^3^ Facultad de Salud Pública y Administración, Universidad Peruana Cayetano Heredia, Lima, Peru; ^4^ Herbert Wertheim School of Public Health and Human Longevity Science, University of California San Diego, La Jolla, CA, USA; ^5^ University of Alabama at Birmingham, Birmingham, AL, USA; ^6^ Scripps Institution of Oceanography, University of California, San Diego, CA, USA

**Keywords:** malaria, human movement, movement ecology, asymptomatic malaria, connectivity

## Abstract

The impact of human population movement (HPM) on the epidemiology of vector-borne diseases, such as malaria, has been described. However, there are limited data on the use of new technologies for the study of HPM in endemic areas with difficult access such as the Amazon. In this study conducted in rural Peruvian Amazon, we used self-reported travel surveys and GPS trackers coupled with a Bayesian spatial model to quantify the role of HPM on malaria risk. By using a densely sampled population cohort, this study highlighted the elevated malaria transmission in a riverine community of the Peruvian Amazon. We also found that the high connectivity between Amazon communities for reasons such as work, trading or family plausibly sustains such transmission levels. Finally, by using multiple human mobility metrics including GPS trackers, and adapted causal inference methods we identified for the first time the effect of human mobility patterns on malaria risk in rural Peruvian Amazon. This study provides evidence of the causal effect of HPM on malaria that may help to adapt current malaria control programmes in the Amazon.

## 1. Introduction

During 2019, 229 million malaria cases worldwide and 409 deaths occurred in 87 endemic countries, which surpassed projections made few years earlier [1]. In Latin America, countries that share the Amazon region account for about 86% of all of the cases in the continent, despite control programmes implemented for several years [1,2]. In Peru, more than 90% of malaria cases are concentrated in the Loreto region, a mainly rural area with mostly no electricity supply, no piped water, accessible only by river, poor housing conditions and high mobility of people between villages [1,3]. Well-identified individual and household-level factors for malaria risk such as the misuse of personal protection measures (e.g. bed nets), knowledge of malaria, occupation household infrastructure, overcrowding, indoor animals and proximity to mosquito breeding sites have been widely described [4–8]. In addition, a number of studies showed the interplay of these individual and household factors with large-scale processes such as climate, deforestation, control programmes and cultural aspects [9–12]. However, most of these previous epidemiological studies relied on static exposures assuming individuals are not moving across different areas. Human population movement (HPM) has been hypothesized as a potential driver of malaria transmission [13,14] but empirical evidence is lacking in the Amazon region.

The HPM between different villages impacts the spread of multiple infectious diseases and it has been observed that HPM is responsible for the spread of vector-borne diseases on scales that exceed the areas covered by their main arthropod vectors [15,16]. In previous studies, standardized (self-reporting) travel questionnaires have been used for the study of HPM [17–19]; however, the lack of space and time granularity collected with these questionnaires often masked the effect of complex travel patterns [20]. In the context of rural and riverine areas such as the Peruvian Amazon, labour activities (e.g. fishermen, loggers and trading) involve long-distance travel through the rivers that connect the whole region, multimodal transportation and multiple intermediate destinations that often include villages that are crossing borders to other countries with heterogeneous endemicity level [21,22]. The particular HPM characteristics in this area require the use of innovative geographical positioning technologies that help the characterization of HPM at a fine scale and with greater precision [18,23].

Previous studies described multiple options to collect data for the study of HPM and its role in disease transmission, such as the use of GPS trackers, cellphone records and participatory mapping (GeoODK) [13,24,25]. These approaches allow the collection of data for spatio-temporal analysis at different levels (neighbourhood to international spatial scale, or daily to seasonal temporal scale). Another approach is based on using data from mobile phones to capture mobility patterns. Data collected with mobile phones generate a large number of records to capture people's mobility that can be then used to quantify HPM patterns. The combination of mobile records with epidemiological surveillance and genetic data improves the estimation of the flows of parasite between localities due to HPM [26]. However, due to privacy concerns, it is not possible to obtain socio-demographic data of the mobile users and in rural areas, such as the Amazon region, the telephone network is scarce and limits the collection of data through these devices [24,27]. Another option to capture population mobility is based on GPS data. GPS data is increasingly being applied to a variety of statistical methods to quantitatively characterize mobility patterns, such as time-weighted spatial averaging (TWSA) approaches (e.g. utilization distribution, kernel density, density ranking) and models based on activity spaces (e.g. daily path area, minimum convex polygon, standard deviation ellipse) at a fine spatio-temporal resolution. The integration of socio-demographic data from travel surveys with the accuracy of GPS data trackers would improve the assessment of HPM and quantify its role on the malaria epidemiology in the Peruvian Amazon. In a previous study [13], we considered only 20 participants with GPS and movement ecology methods to describe the mobility between infected and non-infected participants. For the current paper, we extend the analysis to the whole cohort, combining and comparing two methodologies for the study of HPM (GPS and standardized questionnaires) to investigate the etiological effect of human mobility on malaria.

Another important challenge for the study of the effect of HPM on malaria risk is the complex confounding structure which requires a clear causal inference framework. In the presence of complex confounding structures, methods based on propensity score estimation such as inverse probability of treatment weighting (IPTW) [28] have been proposed to deal with high-dimensional settings while allowing to check and optimize covariate balance to ensure that exposed and unexposed individuals are as similar as possible, thus emulating a target trial [29]. However, such approaches are still underused in the malaria epidemiology literature [30–32] and to the best of our knowledge never applied in the context of HPM.

To bridge this gap in the literature, our study sought to characterize and quantify the mobility patterns and their effects on malaria risk using a densely sampled study in rural Amazonia as a case study. This study leverages a rich human movement dataset (previously reported [13]) with a causal inference framework to estimate the etiological effect of multiple mobility patterns on malaria risk that will provide important insights for malaria control and elimination in rural areas such as the Amazon rainforest region.

## 2. Material and methods

### 2.1. Study design

A population cohort study was conducted to assess the contribution of HPM to the malaria epidemiology in the rural Peruvian Amazon. All inhabitants aged 18 years or older were invited to participate and upon signed consent were followed for two months with weekly measurements. Socio-demographic and epidemiological data were collected at census (week 0), parasitological surveys (weeks 1 and 8) and follow-up surveys (weeks 2 to 7). In addition, a sub-cohort was conducted with 20 selected inhabitants of the main cohort who were given a three-dimensional-printed GPS tracker developed for the study, described elsewhere [13] during the last four weeks of the study (weeks 5 to 8). Malaria infection status was diagnosed weekly by molecular testing. Finger prick blood samples were taken from all participants during the parasitological surveys regardless of their symptoms or travel status. During the follow-up surveys samples were collected if at least one of the following three conditions occurred: (i) the participant reported a travel outside the village the last week, (ii) the GPS tracker recorded travel outside the village (for the sub-cohort participants), and (iii) the participant presented clinical symptoms compatible with malaria.

### 2.2. Study site and population

**Figure 1.**
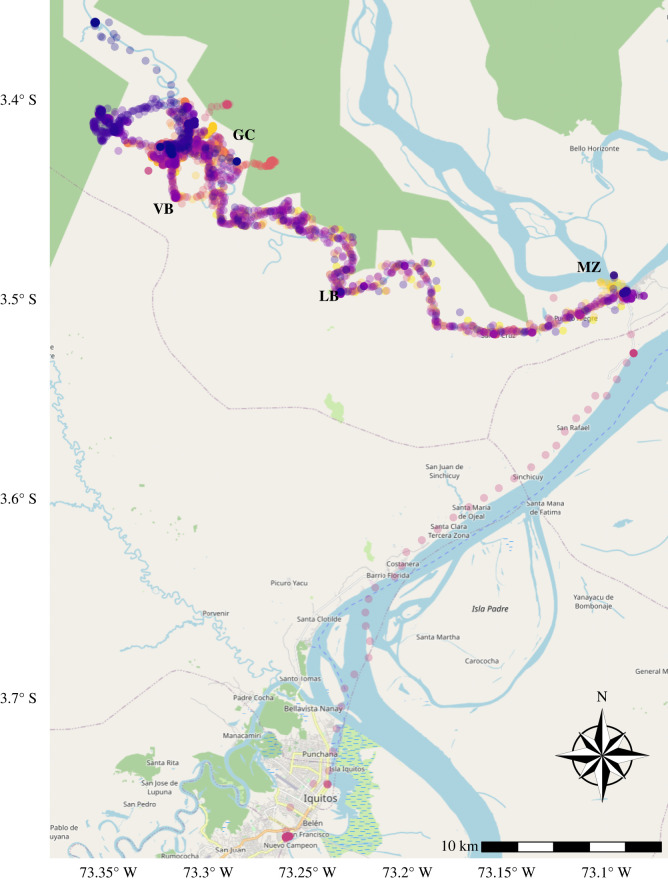
Gamitanacocha in the Peruvian Amazon. Located in the district of Mazan, province of Maynas, region Loreto, Peru. The GPS trackers recorded the trajectories developed by the participants during four weeks. (GC: Gamitanacocha, MZ: Mazan, VB: Visto Bueno, LB: Libertad).

This study was conducted in Gamitanacocha (3.426° S, 73.318° W), in the Mazan district, Maynas province in the Loreto region (figure 1) with a total population of 92 inhabitants. Gamitanacocha is a community located north of Iquitos, capital of the Loreto. The community is only accessible using boat transportation, which takes approximately 6 h from Iquitos. Gamitanacocha is a community surrounded by dense primary and secondary tropical forest with tropical weather and two marked seasons: rainy season from November to May and a dry season from June to October. This climate is optimal for the development of the primary vector of malaria in the region, *Nyssorhynchus (Anopheles) darlingi* [33], making it one of the villages with the highest risk of malaria [19]. All villagers aged 18 years or older (*N* = 50) were included in this study. The GPS trackers developed for this proof-of-concept study were distributed by purposive sampling of participants, taking into account whether the participant had self-reported a trip outside the community in the last month [13].

### 2.3. Data collection

A census of all inhabitants aged 18 years or older in all households was conducted and included socio-demographic data (age, sex, occupation, migratory status, birthplace, time in community, pregnant, chronic disease, educational level, household structure). A parasitological survey (weeks 1 and 8) was conducted to collect epidemiological and mobility history in the last month for the entire population. In addition, the follow-up survey (weeks 2 to 7) was used to collect epidemiological and mobility data for the last week, including whether the GPS tracker recorded participant's departure from the community. These surveys included data such as place of sleeping during travel, reason for travel, work conducted during travel, travel destination and travel duration. It is important to mention that the difference between the main occupation (census) and the work performed during the travel (follow-up survey) was considered because the study population had the characteristic of having occasional or seasonal jobs that did not necessarily coincide with their main occupation. Capillary blood samples were taken on filter paper from all participants to determine the basal and final infection status (parasitological surveys) and the infection status per week of each participant (follow-up survey). Infection status was determined by microscopy and PCR for *Plasmodium* at the species level [19] because infections can be submicroscopic. In addition, a second blood sample was taken 4 days later to avoid false negatives, because the parasitemia may be undetectable at the beginning of the disease. All this information was collected in both the main cohort and the sub-cohort.

### 2.4. Laboratory procedure

In the case of detection by PCR, Genomic DNA was extracted from dry blood spots of approximately 6 mm^2^ sections using E.Z.N.A. Blood DNA Kit (Omega Bio-tek, USA), according to the manufacturer guidelines with slight modifications—addition of TEN (20 mM Tris-HCl, pH 8.0; 2 mM EDTA, pH 8.0; 0.2 M NaCl) buffer, supplemented with SDS 10% w/v. Subsequent amplification was done by a real-time quantitative PCR (qPCR) method targeting the 18SSU rRNA gene region of the *Plasmodium* species-specific region. Oligonucleotides 5-TAACGAACGAGATCTTAA-3 and 5-GTTCCTCTAAGAAGCTTT-3 were used as primers as reported by Mangold *et al*. [34] and a modified protocol was used including PerfeCta SYBR Green Fast Mix (Quanta Biosciences, MD, USA). Ambiguous diagnostic results were confirmed by using a nested ssPCR method [35].

### 2.5. Statistical analysis

The socio-demographic data of all participants and the characteristics of their households were summarized in proportions for categorical variables, and in median and interquartile range for continuous variables with skewed distribution.

#### 2.5.1. Mobility patterns

For the main cohort, mobility patterns derived from the parasitological and follow-up surveys were described, such as travel frequency, destination and reason for travel, work performed during the travel, number of destinations and travel time. For the sub-cohort, GPS trackers were used to record the total distance covered and total time covered (per participant) to observe their relationship with *Plasmodium* spp. infection. The features of the GPS tracker (hardware architecture, code and performance characteristics) were described elsewhere [13].

The GPS records after being integrated with the data of the surveys, were characterized by a non-parametric Bayesian framework using the *Bayesmove* package in R programming software v. 4.0.3 [36], to accurately characterize the mobility patterns of the sub-cohort [37]. The model consists of a two-step framework, which divides individual tracks into segments (electronic supplementary material, figure S1) and then groups these segments into possible movement patterns called 'latent behavioural states', which, depending on whether their characteristics (in terms of step length-SL and turning angle-TA) have biological plausibility, are finally selected. The detailed procedure can be found in electronic supplementary material, methods 1. To obtain a mobility pattern per individual using these generated behavioural states, a summary of these results was performed. First, the proportion of each behavioural state generated was calculated, and second, the optimal value of the proportion of a behavioural state that predicts *Plasmodium* infection was calculated as a breakpoint to define which behavioural state will be assigned as the mobility pattern. This procedure was performed using a ROC curve.

In addition, we compared the performance of GPS trackers and surveys to identify whether participants leave the village, destinations and number of travels. Community departure identified by the GPS trackers was based on the detection of movement out of a 500 m radius with respect to the village centroid. In the case of GPS tracker travel destinations, they were identified by the presence of GPS records within a buffer (approx. 500 m) of the Mazan riverine communities based on the results of the Bayesmove spatial model.

#### 2.5.2. Mobility patterns as risk factors for malaria infection

Multiple mobility pattern metrics were explored, such as travelled more than four times (TN), travelled to Mazan (TD), travelled for work (TR), use a non-motorized boat for travel (TT), sleep outside during the trip (TSP), travelled to Mazan for more than 24 h (M24), travelled to Mazan for reasons other than work (MnW), which were constructed from the weekly surveys, the displacement and commuting pattern by Bayesmove model (MovT). Since the infection status was tested each week, we selected participants free of infection at week 1 (*N* = 30). From these participants, a positive infection status was determined if the participants developed infection during the study period (the first positive result obtained) and, in addition, we computed the time (in weeks) from enrolment to the first malaria infection.

To determine the effect of multiple mobility patterns while dealing with multiple *a priori* identified confounders, we used inverse probability treatment weighting (IPTW) to minimize the differences in baseline characteristics between the groups for each exposure (mobility pattern). The weights were created from the inverse of the propensity score (PS) if the individual was exposed, and for the unexposed it was the inverse of 1 minus the propensity score [38]. The variables used to create the propensity scores for each mobility pattern were *a priori* selected using a directed acyclic graph (DAG) [39] (electronic supplementary material, figure S2) considering all possible causal paths between each exposure (mobility pattern) and malaria infection. We first optimized the propensity score models (considering multiple functional forms and interactions using identified covariates) by comparing the AIC between models. Once we obtained an optimized propensity score (for each comparison of interest), we then created weights as described above [40]. We then checked the covariate balance between exposure groups using standardized mean differences and love plots. We developed three different models to determine the risk of malaria infection associated with HPM exposures through different incidence-based approaches: (i) log-binomial model to estimate the incidence proportion ratio (IPR), (ii) Poisson model accounting for person-week of follow-up as an offset to estimate the incidence rate ratio (IRR), and (iii) Cox proportional-hazards model to estimate the hazard ratio (HR). All analysis and figures were conducted in R programming software v. 4.0.3 [36].

## 3. Results

### 3.1. Baseline characteristics and infection status

**Figure 2.**
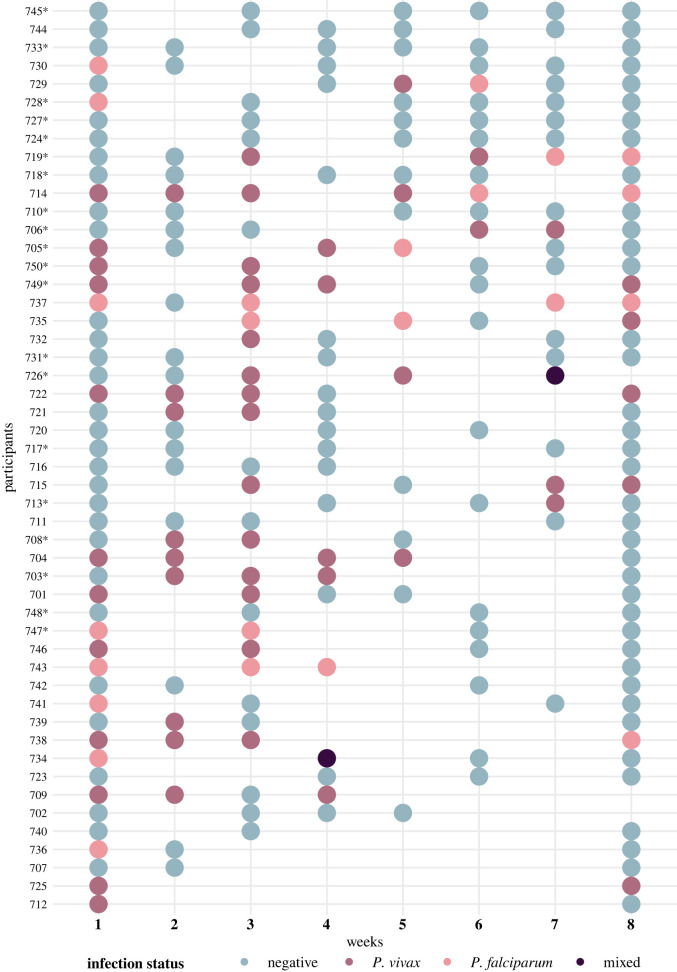
Cases per species detected by PCR weekly of the 50 study participants. Participants who were included in the sub-cohort are indicated with (*). Empty spaces indicate that the participant was not sampled in that week because they did not meet the established criteria for being sampled.

From the 30 participants in the cohort (excluding positives at baseline), the median age was 32 years (IQR = 25–48), the proportion of males and females were the same, and the most frequent occupation was farmer (73%). More than 70% of participants had at least primary education and were literate, only two people had a diagnosis of chronic disease, and 43% of the total population was born in the community. The main reason for migration to Gamitanacocha was family and economic reasons (30% and 23%, respectively). The distribution of socio-economic variables by at least one episode of malaria infection during the study is presented in table 1. The incidence proportion of malaria by PCR in the study was 40%, and the incidence rate was 1 per 10 person-week with a median survival time of two weeks, and the incidence proportion of malaria by microscopy was 17%. Figure 2 shows the distribution of malaria cases by species in the total number of inhabitants of the village during the 8 weeks of the study.

**Table 1.** Baseline characteristics of the study population infection-free at the beginning of the study.

	*N* = 30	*P. vivax*		*P. falciparum*	
		negative	positive	negative	positive
		*n* = 18 (%)	*n* = 12 (%)	*n* = 26 (%)	*n* = 4 (%)
*age* (*years*)*					
18–50	23 (77)	13 (72)	10 (83)	20 (77)	3 (75)
51–65	5 (17)	4 (22)	1 (8.3)	5 (19)	0 (0)
>65	2 (6.7)	1 (5.6)	1 (8.3)	1 (3.8)	1 (25)
*sex*					
male	15 (50)	8 (44)	7 (58)	12 (43)	3 (75)
female	15 (50)	10 (56)	5 (42)	14 (54)	1 (25)
*occupation*					
farmer	22 (73)	14 (78)	8 (67)	19 (73)	3 (75)
housewife	6 (20)	3 (17)	3 (25)	5 (19)	1 (25)
teacher	1 (3.3)	1 (5.6)	0	1 (3.8)	0 (0)
health promoter	1 (3.3)	0 (0)	1 (8.3)	1 (3.8)	0 (0)
*education level*					
none	2 (6.7)	2 (11)	0 (0)	2 (7.7)	0 (0)
primary school	21 (70)	12 (67)	9 (75)	19 (73)	2 (50)
secondary school	5 (17)	2 (11)	3 (25)	3 (12)	2 (50)
higher education	2 (6.7)	2 (11)	0 (0)	2 (7.7)	0 (0)
*literate*					
yes	26 (87)	15 (83)	11 (92)	22 (85)	4 (100)
no	4 (13)	3 (17)	1 (8.3)	4 (15)	0 (0)
*other disease*					
none	28 (93)	16 (89)	12 (100)	24 (92)	4 (100)
anaemia	1 (3.3)	1 (5.6)	0 (0)	1 (3.8)	0 (0)
diabetes	0 (0)	0 (0)	0 (0)	0 (0)	0 (0)
rheumatism	1 (3.3)	1 (5.6)	0 (0)	1 (3.8)	0 (0)
*pregnant*					
yes	2 (13)	0 (0)	2 (40)	2 (14)	0(0)
no	13 (87)	10 (100)	3 (60)	12 (86)	1 (100)
*born in community*					
yes	13 (43)	8 (44)	5 (42)	12 (46)	1 (25)
no	17 (57)	10 (56)	7 (58)	14 (54)	3 (75)
*time in community* (*months*)*					
<12	2 (6.7)	1 (5.6)	1 (8,3)	2 (7.7)	0 (0)
12–24	1 (3.3)	1 (5.6)	0 (0)	1 (3.8)	0 (0)
24–48	4 (13)	2 (11)	2 (17)	4 (15)	0 (0)
48–60	1 (3.3)	1 (5.6)	0 (0)	1 (3.8)	0 (0)
>60	22 (73)	13 (72)	9 (75)	18 (69)	4 (100)
*migration reason*					
none	13 (43)	8 (44)	5 (42)	12 (46)	1 (25)
economic	7 (23)	3 (17)	4 (33)	5 (19)	2 (50)
family	9 (30)	6 (33)	3 (25)	8 (31)	1 (25)
others	1 (3.3)	1 (5.6)	0 (0)	1 (3.8)	0 (0)
*birthplace*					
Gamitanacocha	13 (43)	8 (44)	5 (42)	12 (46)	1 (25)
Marañon	1 (3.3)	1 (5.6)	0 (0)	1 (3.8)	0 (0)
Loreto	1 (3.3)	1 (5.6)	0 (0)	1 (3.8)	0 (0)
Maynas	12 (40)	6 (33)	6 (50)	9 (35)	3 (75)
Putumayo	1 (3.3)	0 (0)	1 (8.3)	1 (3.8)	0 (0)
Ramon Castilla	1 (3.3)	1 (5.6)	0 (0)	1 (3.8)	0 (0)
San Martin	0 (0)	0 (0)	0 (0)	0 (0)	0 (0)
Yurimaguas	1 (3.3)	1 (5.6)	0 (0)	1 (3.8)	0 (0)

*Age and time in community: median (IQR).

Regarding households' characteristics, 86% of the houses use the river as a source of water and 14% use rainwater. Most (86%) households did not have a bathroom, 67% of the households had electricity, and only 14% were fumigated. The floor and walls were made of wood in more than 90% of the households, and the roofs were made of thatch in more than 50%. The characteristics of the total population (*N* = 50) of the village are shown in electronic supplementary material, table S1.

### 3.2. Mobility patterns

In the main cohort (*n* = 30), a total of 132 trips were reported by participants during the study (using standardized questionnaires). Work was the most frequent reason to travel out of the village (42%). Mazan was reported as the most frequent travel destination (36%) and logger the most frequent occupation (table 2). Table 3 shows the mobility patterns and person-weeks for each category of participants (free of infection at baseline); 77% of participants travelled to Mazan at least once, 83% travelled for work at least once, 73% travelled to Mazan not for work at least once, and 53% travelled to Mazan for more than 24 h at least once. The travel characteristics of the total population of the village are presented in electronic supplementary material, table S2.

**Table 2.** Total number of travels of the participants during the whole study.

	*N* = 132	*P. vivax*		*P. falciparum*	
		negative	positive	negative	positive
		*n* = 113(%)	*n* = 19 (%)	*n* = 127 (%)	*n* = 5 (%)
*travel work*					
no work on the travel	91 (69)	77 (68)	14974)	88 (69)	3 (69)
farmer	8 (6.1)	6 (5.3)	2 (11)	7 (5.5)	1 (20)
logger	14 (11)	14 (12)	0 (0)	14 (11)	0 (0)
fisher	8 (6.1)	7 (6.2)	1 (5.3)	8 (6.3)	0 (0)
labourer	3 (2.3)	1 (0.9)	2 (11)	3 (2.4)	0 (0)
others	8 (6.1)	8 (7.1)	0 (0)	7 (5.5)	1 (20)
*travel destination*					
Libertad	7 (5.3)	7 (6.2)	0 (0)	7 (5.5)	0 (0)
Mazan	47 (36)	38 (34)	9 (47)	45 (35)	2 (40)
Visto Bueno	20 (15)	19 (17)	1 (5.3)	19 (15)	1 (20)
others	58 (44)	49 (43)	9 (47)	56 (44)	2 (40)
*travel reason*					
trade	26 (20)	22 (19)	4 (21)	25 (20)	1 (20)
studies	2 (1.5)	2 (1.8)	0 (0)	2 (1.6)	0 (0)
family	21 (16)	16 (14)	5 (26)	20 (16)	1 (20)
health	6 (4.5)	4 (3.5)	2 (11)	6 (4.7)	0 (0)
work	56 (42)	49 (43)	7 (37)	54 (43)	2 (40)
others	21 (16)	20 (18)	1 (5.3)	20 (16)	1 (20)
*travel transportation*					
canoe	13 (9.4)	11 (9.7)	2 (11)	12 (9.4)	1 (20)
motorized boat	109 (83)	92 (81)	17 (89)	105 (83)	4 (80)
others	10 (7.6)	10 (8.8)	0 (0)	10 (7.9)	0 (0)
*travel sleep place*					
no sleep on the travel	73 (55)	65 (58)	8 (42)	69 (54)	4 (80)
house	49 (37)	38 (34)	11 (58)	48 (38)	1 (20)
outside	6 (4.5)	6 (5.3)	0 (0)	6 (4.7)	0 (0)
others	4 (3)	4 (3.5)	0 (0)	4 (3.1)	0 (0)
*travel duration* (*hours*)^a^	40 (9–107)	49 (8–124)	28 (27–51)	49 (9–123)	27 (18–39)

^a^Travel duration (hours): median (IQR).

**Table 3.** Mobility patterns of the participants for the whole study period.

	positive *N* = 12 (%)	population *N* = 30 (%)	pseudo-population	person-week (*N* = 120)
*travelled to Mazan not for work*				
no	2 (17)	8 (27)	33	35
yes	10 (83)	22 (73)	30	85
*travelled to Mazan >24 h*				
no	4 (33)	14 (47)	36	61
yes	8 (67)	16 (53)	21	59
*number of travels (>= 4)*				
no	7 (58)	19 (63)	30	72
yes	5 (42)	11 (37)	25	48
*outside sleep place during travel*				
no	8 (67)	22 (73)	30	90
yes	4 (33)	8 (27)	24	30
*travel in motorized boat*				
no	7 (58)	17 (57)	31	63
yes	5 (42)	13 (43)	30	57
*travel to Mazan*				
no	1 (8.3)	7 (23)	31	33
yes	11 (91.7)	23 (77)	30	87
*travel to work*				
no	1 (8.3)	5 (17)	32	22
yes	11 (91.7)	25 (83)	55	98
*displacement pattern*^a^				
no	3 (38)	8 (53)	15	36
yes	3 (43)	7 (47)	15	32

^a^Per cent for *N* = 15.

**Figure 3.**
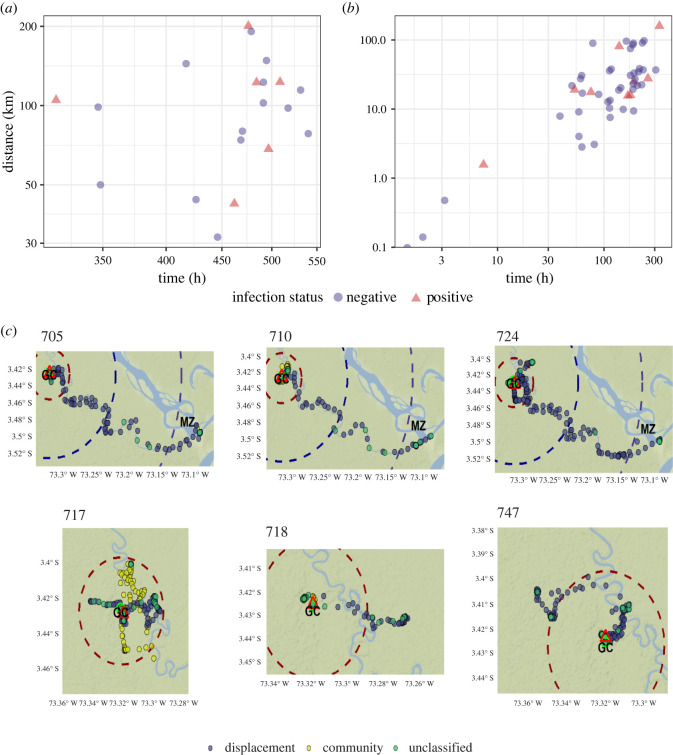
(*a*) Cumulative distance and time travelled by subcohort participants during the whole study by infection status. (*b*) Distance and time travelled weekly by participants in the sub-cohort during the whole study by infection status. (*c*) Trajectories of selected participants outside the village taking into account the type of mobility pattern performed and the distance from Gamitanacocha (red buffer: 3 km, blue buffer: 10 km, violet buffer: 20 km).

For the sub-cohort (*n* = 20), mobility patterns were described in relation to time and distance covered by the participants (using GPS data). Malaria cases increased in those who stayed more time outside the village and travelled more distance, both for the infections summarized per participant (figure 3*a*) and per participant-week of follow-up (figure 3*b*). From the Bayesmove model we obtained probabilities for each GPS record of belonging to a latent behavioural state, showing that the first and second behavioural states grouped the largest number of GPS records. We analysed the characteristics of the two behavioural states with the largest amount of data and identified that the first pattern consisted of long step length movements without a defined turning angle, and another pattern of medium to short step length, with varying turning angles (electronic supplementary material, figure S3). From these results, the first movement pattern identified was labelled as ‘displacement pattern’ and the second, ‘community pattern’. With these mobility patterns, the villages in which participants showed a community pattern, that is, their travel destinations, were identified. The figure 3 shows selected trajectories followed by the participants, with their respective behavioural states. It can also be observed that the trajectories marked by the displacement patterns were heterogeneous, from moving up to a radius of 3 km from Gamitanacocha, to beyond 20 km. Graphics of all participants are in electronic supplementary material, figure S4. The results of the comparison of GPS trackers and standardized questionnaires to detect community departures, number of trips and destinations are shown in electronic supplementary material, table S3.

### 3.3. Effect of mobility patterns on risk of infection by *Plasmodium* spp.

**Figure 4.**
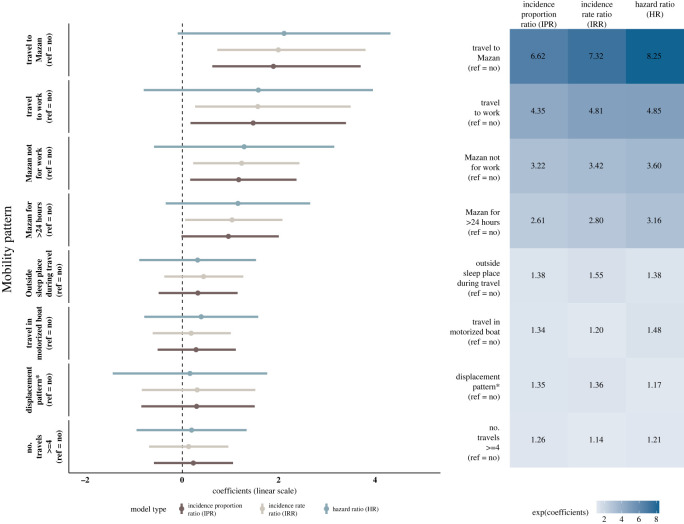
Forest plot of the models for each exposure applying the IP weighting method for each type of model developed. Asterisk (*) represents the mobility metric obtained from the Bayesian spatial model.

Figure 4 shows the results of IPR, IRR and HR (in balanced samples) to estimate the effect of multiple mobility patterns on the risk of malaria infection. Travel to Mazan at least once (IPR: 6.61 [1.87–40.32], IRR: 7.33 [2.07–44.66]), travel to Mazan for other than work (IPR: 3.22 [1.18–10.65], IRR: 3.43 [1.26–11.33]) and travel to work (IPR: 4.35 [1.19–29.76], IRR: 4.79 [1.31–32.76]) were the mobility metrics that increased risk of acquiring a malaria infection in two of the three models considered. Travel to Mazan for more than 24 h (IRR: 2.81 [1.06–7.97]) was the mobility metric shown to be a risk for *Plasmodium* infection in one of the three models. The point estimation of the three models had similar results although for the interval estimates the Cox model less precise. Kaplan–Meier plots (electronic supplementary material, figure S5) show that, although not statistically significant, it takes less time for the exposed group (for each mobility pattern) to reach 50% infection compared with the non-exposed.

## 4. Discussion

The estimation of the etiological effect of human population mobility on malaria risk has been elusive in rural areas in Latin America where moderate-to-high malaria transmission occurs. By using a densely sampled population cohort, this study highlighted the elevated malaria transmission in a riverine community of the Peruvian Amazon. We also found that the high connectivity between Amazon communities for reasons such as work, trading or family plausibly sustain such transmission levels. Finally, by using multiple human mobility metrics including GPS-trackers and causal inference methods we identified the effect of human mobility patterns on malaria risk in rural Peruvian Amazon.

This study area was previously characterized as a moderate transmission setting [41]; however, this is the first report of hyper-endemicity levels comparable to some hyper-endemic areas of sub-Saharan Africa (SSA) such as Guinea (43.9%), Mozambique (38.3%) and Ivory Coast (41.5%) [42]. Importantly, a high level of asymptomatic malaria cases (approx. 88%) has been previously reported in the villages in the Mazan basin, which potentially underestimates actual transmission in the area [35]. In this study, a high detection of infections was accounted by sampling the population twice a week, highlighting the importance of prospective surveillance of malaria. This is particularly important in populations with low parasite densities or close to the limit of detection of diagnostic methods due to the fitness of the immune response in consequence to constant infections [43]. The results obtained in this study are comparable to the 54.3% previously obtained in the district of Mazan [33], 40.9% and 31.8% in the village of Libertad [19,44], Primero de Enero village with 44.4% [19] and in villages that belong to other river basins, such as the Napo river basin, where the Urco Miraño village had a prevalence of 30.7% [19,44]. Further studies are needed to determine the generalizability of the results of this study to the entire Loreto region, mainly due to the high heterogeneity in malaria endemicity and HPM patterns.

This study highlights the importance of considering population mobility when designing strategies to reduce malaria risk. For example, environmental interventions such as vegetation clearance or modification of river boundaries to remove mosquito breeding sites may target areas characterized with higher population mobility patterns. This high transmission observed is possibly fuelled by the high connectivity between communities in the Amazon due to highly mobile populations. In this study, all participants have left the community at least once during the study period (two months). We determined the mobility of the participants through self-reporting travel questionnaires and the use of GPS trackers. By using questionnaires, we determined that travel reasons of the inhabitants of Gamitanacocha were mainly due to work, followed by trading and family. Collecting mobility data from both questionnaires and GPS are complementary, and here we showed how such data can be combined to study the effect of HPM patterns on malaria risk. We, therefore, encourage future studies to rely on diverse sources of data collection. Furthermore, collecting survey data regarding travel reasons has been shown to limit the ‘residential’ effect fallacy [45]. These results are consistent with those reported in Brazil [18,22], where labour was the third cause of travel; however, these reports were collected from surveys in rural and urban areas, where workplaces may be in the same locality. In our study, logging was the most frequent work. Despite the community being surrounded by primary and secondary tropical forest, which allows the development of this activity, long distances were recorded to logging areas where high human-biting behaviours of mosquitoes were previously reported [35]. Another important factor is the seasonality of both vector and work-related travel. This study was conducted during the rainy season when the malaria vector has favourable climatic conditions to develop. In addition, the activities carried out by people are also conditioned by seasonality since there are activities that are carried out on a daily (trading, fishing), periodical (tourism, mining, logging) or long-term (migration, colonization) basis [46]. This connectivity between communities has also been inferred through genetic analysis of parasite populations found in the communities of the same watershed. The level of genetic similarity between parasites sampled in the Mazan watershed indicated an intense flow of parasites between communities, highly compatible with human populations with frequent mobility between them [14].

In this study, we analysed GPS data—to objectively characterize the HPM among villagers—with a Bayesian behavioural state approach. This analysis revealed that there are at least two mobility behaviours in the study participants. We were able to differentiate travel trajectories through the river network from travel in communities or work zones within the forest or forest fringes. It is important to highlight that previous studies reported that these displacements in different communities or forest areas put people at great risk (twofold higher) of getting a malaria infection, because the vector prefers peridomestic areas and the forest fringe [47]. This suggests that interventions to prevent malaria should pay particular attention not only to local transmission but HPM as a key mechanism in moderate-to-high transmission settings. Additionally, we were able to identify the communities most frequented by participants, which for 2018, the Regional Health Direction of Loreto reported annual parasitic indices (API per 10,000 habitants) of 403.4 and 537.5 for Libertad and Visto Bueno villages, 138.8 for Santa Cruz village, 51.9 for Puerto Alegre and 11.6 for Mazan village. Although Mazan does not present high API levels, the malaria risk obtained by the models may be due to these villages, for their location in the basin, are transitory stops on the way to the final destination, which is Mazan. Rich GPS tracking data were leveraged by movement ecology methods such as utilization distribution or kernel density, and other *post hoc* analyses such as step selection functions (SSF), calculation of activity budgets, or behaviour-specific measures of landscape resistance [13,48,49] in the context of non-human animals; however, a body of studies examining these methods for infectious diseases epidemiology are still scarce. Moreover, the fine-scale characterization of HPM obtained in this study is consistent with that described elsewhere [50,51], which gives it an advantage over more commonly used methodologies such as telephone records or self-report surveys. We should also highlight that, in this study, low-cost open-source GPS devices were used [13], which overcomes the logistical limitation that is usually present when researchers want to implement HPM studies at this level of resolution. It is also important to mention that, in this study, the surveys had highly comparable results to the GPS trackers for the detection of community departures by participants. This is possibly because the surveys—like the malaria tests—were conducted on a weekly basis during the whole study, decreasing possible recall bias due to participant omission.

We determined that travel to Mazan city (approx. 3 km from study area) increases the risk of malaria infection, compatible with previous reports that also highlight that the vector is present in the peridomestic zones of the community and in greater frequency in all villages in the watershed of the Mazan river [44,47]. It is also important to note that Mazan is the main city in the entire watershed, so all the other communities interact there for different reasons. Several previous studies have reported the association between travelling for work and the risk of malaria, although due to the characteristics of the type of work carried out in this study (mainly logging) it resulted in a much higher risk compared with the other studies [19,21,22]. From the three types of models developed, the estimates obtained from calculating the IPR and IRR were consistent in the point estimation and precision, in contrast to the HR, which obtained wider CIs, although with the same direction of effect and similar point estimate.

Some limitations in this study must be acknowledged. First, the mobility data from the overall cohort were collected over an eight-week period and the GPS data over a four-week period. This condition would not capture seasonal changes in both mobility patterns and vector development in the area [46]. Future, longitudinal studies are suggested to account for potential seasonal differences in malaria transmission and mobility patterns. Second, the GPS tracker had moments of low battery and had to stop recording data, but the spatial model developed in the study homogenized the recording times in a step prior to the analysis, and the records that could not be smoothed were excluded from the analysis without altering the results. Third, there were participants who were not sampled in all weeks of the study, which could affect the time at which they truly became infected. The weekly sampling strategy was intended to minimize this missing data problem. Fourth, although there were variables that could not be collected, these variables were identified as latent variables through the DAGs and their position as confounders in the causal path were examined. No latent variables were required in the minimum adjustment sets for all DAGs evaluated. Fifth, the study was designed to detect malaria infections during travel and not locally in the community. This could affect the differentiation of whether the participant became infected during travel or in the same community. In addition, we were not able to consider the role of non-travelling asymptomatic individuals in the study area regarding malaria risk. Sixth, personal protection measures (e.g. using of repellent) may act as important effect modifiers. The survey included questions about such individual behaviour measures, but most participants did not answer to these questions. It would be important to investigate the potential protective role of such behaviour measures regarding the effect of mobility and malaria risk. Finally, the small sample size of this study, in addition to the exclusion of participants infected at week 1, may affect the precision of our estimates; however, the different metrics analysed were consistent in direction and magnitude despite the precision estimates.

## 5. Conclusion

This study shows the potential of hyper-endemicity of malaria in riverine communities of the Peruvian Amazon that were supported by high connectivity between villages in the same watershed. We objectively characterized the HPM through the river network and forested areas using GPS tracking data and highlight the mobility patterns that strongly increase the risk of malaria in the area. Finally, this study provides evidence of the etiological effect of human mobility on the risk of malaria that may help tailoring current malaria control strategies in areas with moderate-to-high malaria transmission such as the Amazon rainforest.

## Data Availability

The data and code used to produce the analysis is open access and freely available from https://github.com/healthinnovation/GORGAS and archived in a permanent repository. The data are provided in electronic supplementary material [52].

## References

[1] World Health Organization. 2020 *World malaria report 2020*. Switzerland: OMS. See https://www.who.int/publications-detail-redirect/9789240015791 (accessed 21 March 2021).

[2] Pan American Health Organization. 2020 *Actualización Epidemiológica: Malaria – 10 de junio de 2020*. Washington, DC: OPS/OMS. See https://www.paho.org/es/documentos/actualizacion-epidemiologica-malaria-10-junio-2020 (accessed 31 March 2021).

[3] Montenegro CC, Bustamante-Chauca TP, Pajuelo Reyes C, Bernal M, Gonzales L, Tapia-Limonchi R, Tejedo JR, Chenet SM. 2021 *Plasmodium falciparum* outbreak in native communities of Condorcanqui, Amazonas, Perú. Malar. J. **20**, 88. (10.1186/s12936-021-03608-2) 33579285 PMC7880654

[4] Nahum A et al. 2010 Malaria incidence and prevalence among children living in a peri-urban area on the coast of Benin, West Africa: a longitudinal study. Am. J. Trop. Med. Hyg. **83**, 465-473. (10.4269/ajtmh.2010.09-0611) 20810805 PMC2929036

[5] Gahutu J-B et al. 2011 Prevalence and risk factors of malaria among children in southern highland Rwanda. Malar. J. **10**, 134. (10.1186/1475-2875-10-134) 21592380 PMC3121650

[6] Ayele DG, Zewotir TT, Mwambi HG. 2012 Prevalence and risk factors of malaria in Ethiopia. Malar. J. **11**, 195. (10.1186/1475-2875-11-195) 22691364 PMC3473321

[7] Ferrari G, Ntuku HMT, Ross A, Schmidlin S, Kalemwa DM, Tshefu AK, Lengeler C. 2016 Identifying risk factors for *Plasmodium* infection and anaemia in Kinshasa, Democratic Republic of Congo. Malar. J. **15**, 362. (10.1186/s12936-016-1412-5) 27417676 PMC4946241

[8] Essendi WM, Vardo-Zalik AM, Lo E, Machani MG, Zhou G, Githeko AK, Yan G, Afrane YA. 2019 Epidemiological risk factors for clinical malaria infection in the highlands of Western Kenya. Malar. J. **18**, 211. (10.1186/s12936-019-2845-4) 31234879 PMC6591804

[9] Ikeda T, Behera SK, Morioka Y, Minakawa N, Hashizume M, Tsuzuki A, Maharaj R, Kruger P. 2017 Seasonally lagged effects of climatic factors on malaria incidence in South Africa. Sci. Rep. **7**, 2458. (10.1038/s41598-017-02680-6) 28555071 PMC5447659

[10] Bozcal E, Eldem V, Aydemir S, Skurnik M. 2018 The relationship between phylogenetic classification, virulence and antibiotic resistance of extraintestinal pathogenic *Escherichia coli* in İzmir province, Turkey. PeerJ. **6**, e5470. (10.7717/peerj.5470) 30155366 PMC6110251

[11] MacDonald AJ, Mordecai EA. 2019 Amazon deforestation drives malaria transmission, and malaria burden reduces forest clearing. Proc. Natl Acad. Sci. USA **116**, 22 212-22 218. (10.1073/pnas.1905315116) PMC6825316 31611369

[12] Essé C et al. 2008 Social and cultural aspects of ‘malaria’ and its control in central Côte d'Ivoire. Malar. J. **7**, 224. (10.1186/1475-2875-7-224) 18973663 PMC2588631

[13] Carrasco-Escobar G et al. 2020 Open-source 3D printable GPS tracker to characterize the role of human population movement on malaria epidemiology in river networks: a proof-of-concept study in the Peruvian Amazon. Front. Public Health. **8**, 526468. (10.3389/fpubh.2020.526468) 33072692 PMC7542225

[14] Manrique P et al. 2019 Microsatellite analysis reveals connectivity among geographically distant transmission zones of *Plasmodium vivax* in the Peruvian Amazon: a critical barrier to regional malaria elimination. PLoS Negl Trop. Dis. **13**, e0007876. (10.1371/journal.pntd.0007876) 31710604 PMC6874088

[15] Bradley J, Monti F, Rehman AM, Schwabe C, Vargas D, Garcia G, Hergott D, Riloha M, Kleinschmidt I. 2015 Infection importation: a key challenge to malaria elimination on Bioko Island, Equatorial Guinea. Malar. J. **14**, 46. (10.1186/s12936-015-0579-5) 25651929 PMC4323054

[16] Wesolowski A, Eagle N, Tatem AJ, Smith DL, Noor AM, Snow RW, Buckee CO. 2012 Quantifying the impact of human mobility on malaria. Science **338**, 267-270. (10.1126/science.1223467) 23066082 PMC3675794

[17] Maheu-Giroux M, Casapía M, Gyorkos TW. 2011 On the validity of self-reports and indirect reports to ascertain malaria prevalence in settings of hypoendemicity. Soc. Sci. Med. **72**, 635-640. (10.1016/j.socscimed.2010.12.007) 21257247

[18] Gomes MFC, Codeço CT, Bastos LS, Lana RM. 2020 Measuring the contribution of human mobility to malaria persistence. Malar. J. **19**, 1-2. (10.1186/s12936-019-3075-5) 33176792 PMC7659106

[19] Carrasco-Escobar G et al. 2017 Micro-epidemiology and spatial heterogeneity of *P. vivax* parasitaemia in riverine communities of the Peruvian Amazon: a multilevel analysis. Sci. Rep. **7**, 8082. (10.1038/s41598-017-07818-0) 28808240 PMC5556029

[20] Smith DL et al. 2014 Recasting the theory of mosquito-borne pathogen transmission dynamics and control. Trans. R Soc. Trop. Med. Hyg. **108**, 185-197. (10.1093/trstmh/tru026) 24591453 PMC3952634

[21] Gunderson AK et al. 2020 Malaria transmission and spillover across the Peru–Ecuador border: a spatiotemporal analysis. Int. J. Environ. Res. Public Health **17**, 7434. (10.3390/ijerph17207434) 33066022 PMC7600436

[22] Carrasco-Escobar G, Miranda-Alban J, Fernandez-Miñope C, Brouwer KC, Torres K, Calderon M, Gamboa D, Llanos-Cuentas A, Vinetz JM. 2017 High prevalence of very-low *Plasmodium falciparum* and *Plasmodium vivax* parasitaemia carriers in the Peruvian Amazon: insights into local and occupational mobility-related transmission. Malar. J. **16**, 415. (10.1186/s12936-017-2063-x) 29037202 PMC5644076

[23] Marasinghe DH, Cheaveau J, Meatherall B, Kuhn S, Vaughan S, Zimmer R, Pillai DR. 2020 Risk of malaria associated with travel to malaria-endemic areas to visit friends and relatives: a population-based case-control study. CMAJ Open **8**, E60-E68. (10.9778/cmajo.20190070) PMC6996033 31992561

[24] Carrasco-Escobar G, Castro MC, Barboza JL, Ruiz-Cabrejos J, Llanos-Cuentas A, Vinetz JM, Gamboa D. 2019 Use of open mobile mapping tool to assess human mobility traceability in rural offline populations with contrasting malaria dynamics. PeerJ. **7**, e6298. (10.7717/peerj.6298) 30697487 PMC6346981

[25] Tatem AJ et al. 2014 Integrating rapid risk mapping and mobile phone call record data for strategic malaria elimination planning. Malar. J. **13**, 52. (10.1186/1475-2875-13-52) 24512144 PMC3927223

[26] Chang H-H et al. 2019 Mapping imported malaria in Bangladesh using parasite genetic and human mobility data. Elife **8**, e43481. (10.7554/eLife.43481) 30938289 PMC6478433

[27] Wesolowski A et al. 2014 Quantifying travel behavior for infectious disease research: a comparison of data from surveys and mobile phones. Sci. Rep. **4**, 5678. (10.1038/srep05678) 25022440 PMC4894426

[28] Moore KL, Neugebauer R, van der Laan MJ, Tager IB. 2012 Causal inference in epidemiological studies with strong confounding. Stat. Med. **31**, 1380-1404. (10.1002/sim.4469) 22362629 PMC4113478

[29] Hernán MA, Robins JM. 2016 Using big data to emulate a target trial when a randomized trial is not available. Am. J. Epidemiol. **183**, 758-764. (10.1093/aje/kwv254) 26994063 PMC4832051

[30] Dwomoh D, Agyabeng K, Agbeshie K, Incoom G, Nortey P, Yawson A, Bosomprah S. 2020 Impact evaluation of the free maternal healthcare policy on the risk of neonatal and infant deaths in four sub-Saharan African countries: a quasi-experimental design with propensity score kernel matching and difference in differences analysis. BMJ Open **10**, e033356. (10.1136/bmjopen-2019-033356) PMC7232624 32414818

[31] Cates JE et al. 2017 Malaria, malnutrition, and birthweight: a meta-analysis using individual participant data. PLoS Med. **14**, e1002373. (10.1371/journal.pmed.1002373) 28792500 PMC5549702

[32] Leopold SJ, Watson JA, Jeeyapant A, Simpson JA, Phu NH, Hien TT, Day NP, Dondorp AM, White NJ. 2019 Investigating causal pathways in severe falciparum malaria: a pooled retrospective analysis of clinical studies. PLoS Med. **16**, e1002858. (10.1371/journal.pmed.1002858) 31442221 PMC6707545

[33] Moreno-Gutierrez D et al. 2018 Effectiveness of a malaria surveillance strategy based on active case detection during high transmission season in the Peruvian Amazon. Int. J. Environ. Res. Public Health **15**, 2670. (10.3390/ijerph15122670) 30486449 PMC6314008

[34] Mangold KA, Manson RU, Koay ESC, Stephens L, Regner M, Thomson Jr RB, Peterson LR, Kaul KL. 2005 Real-time PCR for detection and identification of *Plasmodium* spp. J. Clin. Microbiol. **43**, 2435-2440. (10.1128/JCM.43.5.2435-2440.2005) 15872277 PMC1153761

[35] Parker BS et al. 2013 Hyperendemic malaria transmission in areas of occupation-related travel in the Peruvian Amazon. Malar. J. **12**, 178. (10.1186/1475-2875-12-178) 23724869 PMC3673823

[36] R Core Team. 2013 *R: a language and environment for statistical computing*. Vienna, Austria: R Foundation for Statistical Computing. See https://www.r-project.org/.

[37] Cullen J. 2020 joshcullen/bayesmove [Internet]. See https://github.com/joshcullen/bayesmove (accessed 8 February 2021).

[38] Kaura A et al. 2020 Invasive versus non-invasive management of older patients with non-ST elevation myocardial infarction (SENIOR-NSTEMI): a cohort study based on routine clinical data. Lancet **396**, 623-634. (10.1016/S0140-6736(20)30930-2) 32861307 PMC7456783

[39] Textor J, van der Zander B, Gilthorpe M, Liskiewicz M, Ellison G. 2016 Robust causal inference using directed acyclic graphs: the R package 'dagitty'. Inter. J. Epi. **45**, 1887-1894. . 10.1093/ije/dyw341 28089956

[40] Somers EC et al. 2021 Tocilizumab for treatment of mechanically ventilated patients with COVID-19. Clin. Infect. Dis. **73**, e445-e454. (10.1093/cid/ciaa954) 32651997 PMC7454462

[41] Torres K, Alava F, Soto-Calle V, Llanos-Cuentas A, Rodriguez H, Llacsahuanga L, Gamboa D, Vinetz J. 2020 Malaria situation in the Peruvian Amazon during the COVID-19 pandemic. Am. J. Trop. Med. Hyg. **103**, 1773-1776. (10.4269/ajtmh.20-0889) 32885776 PMC7646770

[42] Papaioannou I, Utzinger J, Vounatsou P. 2019 Malaria-anemia comorbidity prevalence as a measure of malaria-related deaths in sub-Saharan Africa. Sci. Rep. **9**, 1-9. (10.1038/s41598-019-47614-6) 31383881 PMC6683112

[43] Roshanravan B et al. 2003 Endemic malaria in the Peruvian Amazon region of Iquitos. Am. J. Trop. Med. Hyg. **69**, 45-52. (10.4269/ajtmh.2003.69.45) 12932096

[44] Rosas-Aguirre A et al. 2021 Integrating parasitological and entomological observations to understand malaria transmission in riverine villages in the Peruvian Amazon. J. Infect. Dis. **223**, S99-S110. (10.1093/infdis/jiaa496) 33906225 PMC8079135

[45] Chaix B, Duncan D, Vallée J, Vernez-Moudon A, Benmarhnia T, Kestens Y. 2017 The «residential» effect fallacy in neighborhood and health studies: formal definition, empirical identification, and correction. Epidemiol. Camb. Mass. **28**, 789-797. (10.1097/EDE.0000000000000726) 28767516

[46] Martens P, Hall L. 2000 Malaria on the move: human population movement and malaria transmission. Emerg. Infect. Dis. **6**, 103-109. (10.3201/eid0602.000202) 10756143 PMC2640853

[47] Saavedra MP et al. 2019 Higher risk of malaria transmission outdoors than indoors by Nyssorhynchus darlingi in riverine communities in the Peruvian Amazon. Parasit. Vectors **12**, 374. (10.1186/s13071-019-3619-0) 31358033 PMC6664538

[48] Fornace KM et al. 2019 Local human movement patterns and land use impact exposure to zoonotic malaria in Malaysian Borneo. Elife **8**, e47602. (10.7554/eLife.47602) 31638575 PMC6814363

[49] Cullen JA, Poli CL, Fletcher RJ, Valle D. 2021 Identifying latent behavioral states in animal movement with M4, a non-parametric Bayesian method. *bioRxiv*. 2020.11.05.369702. (10.1101/2020.11.05.369702)

[50] Searle KM, Lubinda J, Hamapumbu H, Shields TM, Curriero FC, Smith DL, Thuma PE, Moss WJ. 2017 Characterizing and quantifying human movement patterns using GPS data loggers in an area approaching malaria elimination in rural southern Zambia. R. Soc. Open Sci. **4**, 170046. (10.1098/rsos.170046) 28573009 PMC5451810

[51] Ueno TMRL, Lima LNGC, Sardinha DM, Rodrigues YC, Souza Hd, Teixeira PR, Guimarães RJ, Lima KV, Ventura AM. 2021 Socio-epidemiological features and spatial distribution of malaria in an area under mining activity in the Brazilian Amazon region. Int. J. Environ. Res. Public Health. **18**, 10384. (10.3390/ijerph181910384) 34639684 PMC8507758

[52] Carrasco-Escobar G, Matta-Chuquisapon J, Manrique E, Ruiz-Cabrejos J, Barboza JL, Wong D, Henostroza G, Llanos-Cuentas A, Benmarhnia T. 2022 Quantifying the effect of human population mobility on malaria risk in the Peruvian Amazon. Figshare. (10.6084/m9.figshare.c.6080902) PMC9297009 35875474

